# The effect of body mass index on the outcomes of cementless medial mobile-bearing unicompartmental knee replacements

**DOI:** 10.1007/s00167-021-06549-0

**Published:** 2021-04-17

**Authors:** Hasan Raza Mohammad, Stephen Mellon, Andrew Judge, Christopher Dodd, David Murray

**Affiliations:** 1 Nuffield Department of Orthopaedics, Rheumatology and Musculoskeletal Sciences, University of Oxford, Nuffield Orthopaedic Centre, Oxford, OX3 7LD UK; 2 Musculoskeletal Research Unit, Bristol Medical School, University of Bristol, Level 1 Learning and Research Building, Southmead Hospital, Westbury-On-Trym, Bristol, BS10 5NB UK; 3 Nuffield Orthopaedic Centre, Windmill Road, Oxford, OX3 7LD UK

**Keywords:** Body mass index, Cementless fixation, Mid-long term outcomes, Unicondylar knee replacement

## Abstract

**Purpose:**

Given an increasingly overweight population, unicompartmental knee replacements (UKRs) are being performed in patients with higher body mass indices (BMIs). There are concerns that cemented fixation will not last. Cementless fixation may offer a solution, but the long term results in different BMI groups has not been assessed. We studied the effect of BMI on the outcomes of cementless UKRs.

**Methods:**

A prospective cohort of 1000 medial cementless mobile-bearing UKR with a mean follow up of 6.6 years (SD 2.7) were analysed. UKRs were categorised into four BMI groups: (1) ≥ 18.5 to < 25 kg/m^2^ (normal), (2) 25 to < 30 kg/m^2^ (overweight), (3) 30 to < 35 kg/m^2^ (obese class 1) and (4) ≥ 35 kg/m^2^ (obese class 2). Implant survival was assessed using endpoints reoperation and revision. Functional outcomes were assessed.

**Results:**

Ten-year cumulative revision rate for the normal (*n* = 186), overweight (*n* = 434), obese class 1 (*n* = 213) and obese class 2 (*n* = 127) groups were 1.8% (CI 0.4–7.4), 2.6% (CI 1.3–5.1), 3.8% (CI 1.5–9.2) and 1.7% (CI 0.4–6.8) with no significant differences between groups (*p* = 0.79). The 10-year cumulative reoperation rates were 2.7% (CI 0.8–8.2), 3.8% (CI 2.2–6.6), 5.2% (CI 2.5–10.7) and 1.7% (CI 0.4–6.8) with no significant differences between groups (*p* = 0.44). The 10-year median Oxford Knee Score were 43.0, 46.0, 44.0 and 38.0 respectively.

**Conclusion:**

Cementless mobile-bearing UKR has low 10-year reoperation and revision rates across in all BMI groups, and there are no significant differences between the groups. Although higher BMI groups had slightly worse functional outcomes, the improvement in function compared to preoperatively  tended to be better. This study suggests that BMI should not be considered a contraindication for the cementless mobile-bearing UKR.

## Introduction

The two main established treatments for end-stage medial compartment osteoarthritis and avascular necrosis of the knee are total knee replacement (TKR) and unicompartmental knee replacement (UKR) [26]. UKR offers several advantages over TKR but has higher revision rates. In national joint registries UKR revision rates are much higher than those for TKRs whereas in some cohort series they are similar [6, 19, 40]. This is primarily because there is variability in the indications and contraindications for UKR with some being implanted in situations where there is a high failure rate [13, 16, 22].

The number of knee replacements performed annually is rapidly increasing with a greater proportion of overweight and obese patients now needing joint replacements and this is only projected to increase with time [8, 18]. Several clinical commissioning groups in the United Kingdom (UK) currently ration knee replacement surgery based on BMI in part because of  concerns over implant survival [23, 34].

It is well known that raised BMI is associated with increased surgical site infections, thromboembolic events, worse functional outcomes, pain and revision for TKR surgery [2, 20, 38]. The rates of aseptic loosening for TKRs has been reported to be twice as high in obese patients [1, 2]. This creates concern for UKR given its revision rates are already higher than TKR in the joint registries [4, 26, 37]. However, studies of the effect of BMI on UKR outcomes have given conflicting results [7, 23, 27, 32, 42]. There remains concern that cementless implants will not supply adequate fixation for those with elevated BMIs given these patients generally apply greater loads to the bone-prosthesis interface [39] and there is no cement to augment primary stability postoperatively.

The most commonly used UKR is the Oxford UKR (Zimmer Biomet, Swindon, UK), which is implanted via a minimally invasive approach [26]. The cementless Oxford was introduced in 2004 and has a coating of calcium hydroxyapatite and porous plasma-sprayed titanium on its surface [10]. Cohort studies and randomised controlled trials (RCTs) have demonstrated a reduced incidence of radiolucencies and similar clinical and functional outcomes compared to the cemented Oxford UKR [14, 30]. However, the effect of BMI on the mid to long term outcomes of the cementless Oxford UKR have not been studied.

The aim of this study is to analyse the effect of BMI on the mid to long term clinical outcomes of the cementless Oxford UKR. In this study, BMI was not considered to be a contraindication to UKR surgery.

## Materials and methods

Between June 2004 and October 2017, 1000 medial cementless Oxford UKRs were performed through a minimally invasive approach by two surgeons involved in the design of the implant using the recommended surgical approach and technique, and the recommended clinical indications [12]. The indications were based on patho-anatomy with the indications being anteromedial osteoarthritis (AMOA) and medial avascular necrosis. Appropriate AMOA cases were those with medial bone on bone arthritis, a functionally intact anterior cruciate ligament and full-thickness cartilage in the lateral compartment as described previously [21]. In this study, BMI was not considered to be a contraindication to UKR surgery.

BMI groups at the time of surgery were categorised a priori as per the World Health Organisation [29] into five groups; (1) Underweight (BMI < 18.5 kg/m^2^), (2) Normal weight (≥ 18.5 to < 25 kg/m^2^), (3) Overweight (BMI ≥ 25 to < 30 kg/m^2^), (4) Obese Class 1 (BMI ≥ 30 to < 35 kg/m^2^) and (5) Obese Class 2 (BMI ≥ 35 kg/m^2^). There were no patients classified as underweight at the time of surgery leaving four analysis groups for comparison.

Patients were prospectively recruited and assessed preoperatively and at 1, 2, 5 and 10 years postoperatively by research physiotherapists independent of the surgical teams taking care of the patients. During the study 44 knees withdrew from regular follow up; 28 knees from patients with poor health, 6 knees from patients going abroad and 10 knees from patients requesting to leave the study. None of the patients who were withdrawn from the study were reported by the NJR as having had a revision. Height and weight data were missing for 40 (4%) UKRs and therefore these knees could not be included in the BMI analyses. From the 960 knees available with BMI data for analysis, 41 knees were lost from patients dying during the study from causes unrelated to surgery, but their implant status was known at the time of death. Eleven deaths occurred in the normal weight group, 17 in the overweight group, 5 in the obese (class 1) group and 8 in the obese (class 2) group.

For the survival analysis failure was defined as revision and reoperation. Revision was defined as the removal, addition or replacement of any implant component as per the joint registries [4, 26, 37]. Revision was further divided into major revision defined as those requiring revision knee replacement components such as stems, wedges and constraint, which are typically used for revising TKR. Reoperation was defined as any further surgical intervention to the knee and included manipulations under anaesthesia, arthroscopies, fracture fixation and all revisions. The advantage of this outcome is the detection of further operations which are not recorded by the joint registries and which from a patient’s point of view are in many ways similar to a revision.

Functional outcomes were assessed at follow up timepoints using; Oxford Knee Score (OKS), American Knee Society Objective Score (AKSS-O), American Knee Society Functional Score (AKSS-F) and the Tegner Activity Score. The AKSS-O was calculated as previously described [21] without deductions if the post-operative alignment was not neutral, as the Oxford UKR does not aim to achieve neutral alignment like TKR, but aims to restore pre-disease alignment [11]. Additionally, the Charnley score, maximum knee flexion and the range of extension were also recorded. The OKS in different Charnley groups is reported narratively. Flexion was recorded as positive values, with hyperextension recorded as negative values. Differences between preoperative and postoperative patient reported outcome measure (PROM) scores were calculated. Both analyses of the differences in PROMs and the OKS in different Charnley groups were not performed at 10 years given limitations in the numbers available for analysis.

Complications or further operations were recorded when they occurred or at each follow-up appointment. Patients who were unable to attend were contacted by post or telephone to obtain the relevant clinical information. Our prospective database is updated in real-time by a full-time data manager with data extracted on 15th March 2020.

### Statistical analysis

To assess implant survival and cumulative failure rate for both reoperation and revision endpoints the Kaplan Meier method was utilised. Differences in implant survival between the BMI groups was tested using the log-rank test.

Continuous variables were described using means, standard deviations (SDs), medians and interquartile ranges (IQRs). Categorical variables were tabulated with absolute frequencies. Continuous PROMs data were not normally distributed and therefore appropriate nonparametric tests were utilised. To analyse differences in PROMs between the different BMI groups the Kruskal–Wallis test was used.

Maximum extension and flexion data were normally distributed and was therefore compared between BMI groups using the one-way analysis of variance. Hyperextension angles were recorded as negative values. The Charnley score was compared between BMI groups using the Chi-squared proportional test.

Statistical analyses were all performed in Stata version 14 (STATA Corp, TX). *p* values of < 0.05 were considered significant with and 95% confidence intervals (CIs) are reported where appropriate.

## Results

Of the 1000 UKRs, 960 had BMI data available and were included in the analysis for this study. Nine hundred and forty-nine knees had a diagnosis of anteromedial osteoarthritis and 11 had spontaneous osteonecrosis of the knee. Seventy-three percent of the cohort were unilateral with the remaining bilateral. Fifty-four percent of the cohort were male knees, the mean age at surgery was 66.2 years (SD 10.0) and mean BMI was 29.1 (SD 5.0). All patients satisfied the recommended indications as described by Goodfellow et al. [9]. The mean follow-up was 6.6 years (SD 2.7) with 68 and 10% of UKRs having a minimum follow up 5 and 10 years respectively. The numbers in each BMI group and their follow up are summarised are summarised in Table 1.

**Table 1:** Baseline descriptive statistics of the cohort

	Normal weight	Overweight	Obese (class 1)	Obese (class 2)
Number of knees	186	434	213	127
Number of knees with minimum 5 years follow up	118	301	144	87
Number of knees with minimum 10 years follow up	21	46	15	15
Mean BMI	23.2 (SD 1.4)	27.5 (SD 1.4)	32.2 (SD 1.4)	38.3 (SD 3.5)
BMI range	18.8–24.9	25.0–29.9	30.0–34.9	35.0–52.7
Mean age	69.1 (SD 10.4)	66.5 (SD 10.1)	64.6 (SD 9.4)	63.6 (SD 8.6)
Sex (proportion male)	0.43	0.64	0.54	0.38
Preop OKS	26.9 (SD 8.4) 27.0 (IQR 12.0)	26.7 (SD 7.5) 27.0 (IQR 11.0)	24.2 (SD 8.5) 24.0 (IQR 11.0)	20.8 (SD 8.8) 20.0 (IQR 12.0)
Preop Tegner	2.3 (SD 0.99) 2.0 (IQR 1.0)	2.6 (SD 1.2) 3.0 (IQR 1.0)	2.3 (SD 1.2) 2.0 (IQR 2.0)	2.0 (SD 1.2) 2.0 (IQR 2.0)
Preop AKSS-O	61.9 (SD 16.8) 63.5 (IQR 29.0)	62.3 (SD 14.5) 60.0 (IQR 17.0)	59.2 (SD 15.8) 60.0 (IQR 22.5)	53.9 (SD 13.8) 54.0 (SD 20.0)
Preop AKSS-F	71.9 (SD 14.8) 70.0 (IQR 20.0)	73.8 (SD 16.8) 70.0 (IQR 27.5)	68.9 (SD 16.9) 70.0 (IQR 20.0)	63.7 (SD 17.8) 65.0 (IQR 15.0)

There were 186 UKRs in the normal weight group, 434 UKRs in the overweight group, 213 UKRs in the obese (class 1) group and 127 UKRs in the obese (class 2) group. The baseline characteristics between the different BMI groups are summarised in Table 1. Higher BMI groups had slightly lower mean ages. Overweight and obese (class 1) groups had the greatest proportion of male patients. Normal and overweight groups had slightly higher preoperative scores than the obese groups.

**Fig. 1:**
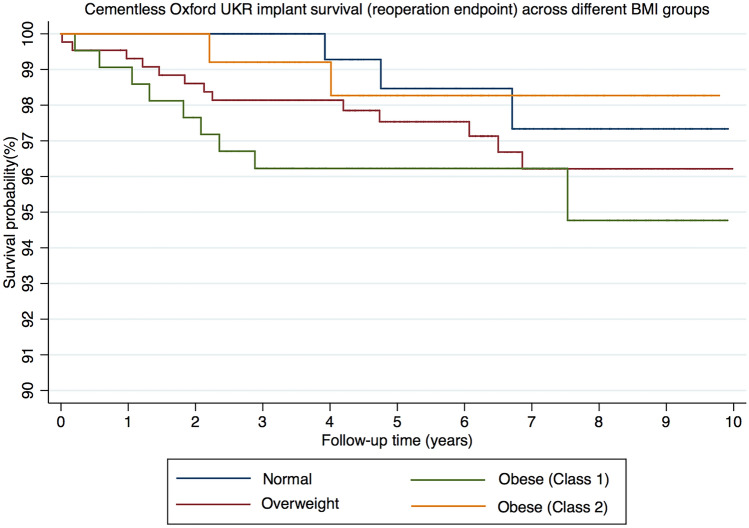
Kaplan–Meier graph of cementless Oxford UKR implant survival (reoperation endpoint) across different BMI groups

In the entire cohort, there were 28 reoperations at a mean of 3.2 years (SD 2.6). The details of the reoperations in the different age groups are summarised in Table 2. Using reoperation as an endpoint the 5 and 10-year implant survival of the normal weight group was 98.5% (CI 94.0–99.6) and 97.3% (CI 91.8–99.2), for the overweight group was 97.5% (CI 95.5–98.7) and 96.2% (CI 93.4–97.8), for the obese (class 1) group was 96.2% (CI 92.6–98.1) and 94.8% (CI 89.3–97.5) and for the obese (class 2) group was 98.3% (CI 93.2–99.6) and 98.3% (CI 93.2–99.6) (Fig. 1). There were no significant differences in implant survival (reoperation) between groups (*p* = 0.44).

**Table 2:** Details of reoperations and revisions in each BMI group

BMI group	Number of reoperations	Number of revisions	Details of reoperations/revisions	Indication for surgery
Normal	4	3	1 bearing exchange	Bearing dislocation
			1 lateral UKR	Tibial avascular necrosis
			1 TKR	Disease progression
			1 arthroscopy	Lateral meniscal tear
Overweight	13	9	1 TKR	Pain
			3 lateral UKRs	Disease progression
			2 arthroscopies	1 for Loose body and 1 for Swelling
			4 bearing exchange	Bearing dislocation
			1 washout debridement and closure	Wound dehiscence
			1 open washout	Suspected infection
			1 tibial component revision	Pain
Obese (class 1)	9	6	2 arthroscopies	Pain
			3 bearing exchange	Bearing dislocation
			1 cemented femoral component	Femoral component loosening
			1 lateral UKR	Disease progression
			1 patellofemoral replacement	Pain
			1 aspiration and MUA	Pain and intermittent swelling/stiffness
Obese (class 2)	2	2	2 TKR	1 Lateral tibial fracture* and 1 Disease progression*

*Major revision

**Fig. 2:**
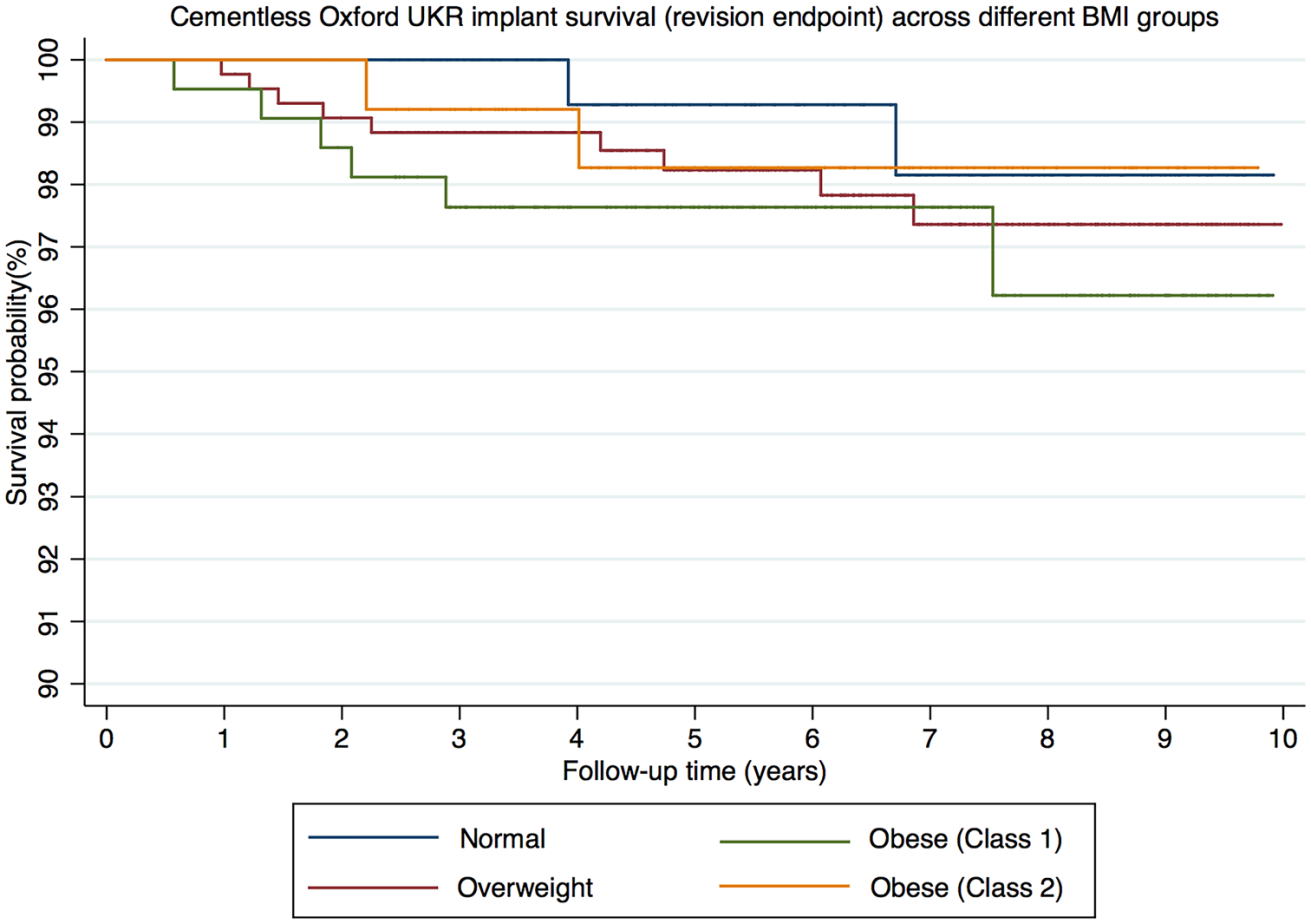
Kaplan–Meier graph of cementless Oxford UKR implant survival (revision endpoint) across different BMI groups

From the 28 reoperations, 20 met the definition of implant revisions at mean 3.7 years (SD 2.7). The details of the revisions in the different age groups are summarised in Table 2. Using revision as an endpoint the 5 and 10-year implant survival of the normal group was 99.3% (CI 95.0–99.9) and 98.2% (CI 92.6–99.6), for the overweight group was 98.2% (CI 96.3–99.2) and 97.4% (CI 94.9–98.7), for the obese (class 1) was 97.6% (CI 94.4–99.0) and 96.2% (CI 90.8–98.5) and for the obese (class 2) group was 98.3% (CI 93.2–99.6) and 98.3% (CI 93.2–99.6) (Fig. 2). There were no significant differences in implant survival (revision) between groups (*p* = 0.79).

From the 20 revisions, 2 met the definition of major revision [both in the obese (class 2) group]. One knee was converted to a TKR with a stemmed tibial implant following a lateral tibial plateau fracture after a fall and one knee was converted to TKR with tibial stem for lateral disease progression.

**Fig. 3:**
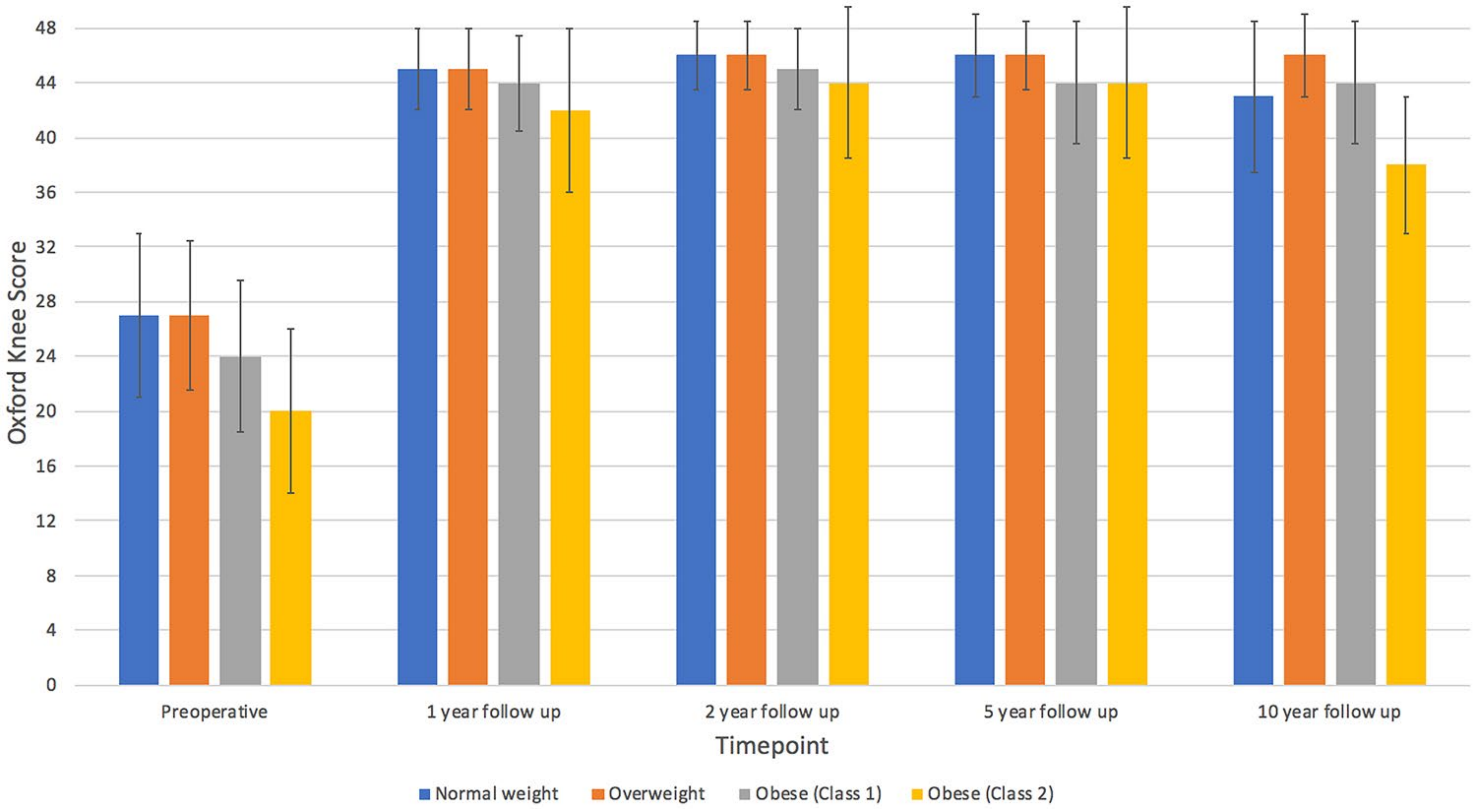
Bar chart of the median OKS at different time points in the different BMI groups. Error bars represent the interquartile range

The mean and median postoperative OKS, AKSS-O, AKSS-F and Tegner scores at 1, 2, 5 and 10 years improved in all age groups (Table 3) compared to each group’s respective preoperative PROM scores (Table 1; Fig. 3).

**Table 3:** Post-operative outcomes in the different BMI groups at different time points.

	BMI group				Significance
	Normal	Overweight	Obese (class 1)	Obese (class 2)	*p* value
1-year					
1 year OKS	42.7 (SD 6.1) 45.0 (IQR 6.0)	42.9 (SD 5.8) 45.0 (IQR 6.0)	41.4 (SD 6.8) 44.0 (IQR 7.0)	39.0 (SD 9.5) 42.0 (IQR 12.0)	< 0.001
1 year Tegner	3.1 (SD 1.2) 3.0 (IQR 0.0)	3.3 (SD 1.2) 3.0 (IQR 1.0)	3.0 (SD 1.1) 3.0 (IQR 2.0)	2.6 (SD 1.2) 3.0 (IQR 1.0)	< 0.001
1 year AKSS-O	93.4 (SD 10.3) 95.0 (IQR 7.0)	93.5 (SD 9.3) 95.0 (IQR 7.0)	90.2 (SD 11.7) 94.0 (IQR 8.0)	87.1 (SD 15.7) 94.0 (IQR 14.0)	< 0.001
1 year AKSS-F	87.9 (SD 14.3) 100.0 (IQR 20.0)	87.9 (SD 14.8) 100.0 (IQR 20.0)	84.2 (SD 14.3) 80.0 (IQR 17.5)	77.6 (SD 19.8) 80.0 (IQR 30.0)	< 0.001
1 year Charnley score	28.6% A 42.2% B 29.2% C	26.6% A 50.0% B 23.4% C	16.3% A 51.2% B 32.5% C	16.4% A 40.4% B 43.3% C	0.003
1 year max flexion (°)	132.4 (SD 10.5) 134.5 (IQR 14.0)	129.3 (SD 9.1) 130.0 (IQR 9.0)	124.9 (SD 8.4) 125.0 (IQR 11.0)	119.2 (SD 13.2) 120.0 (IQR 15.0)	< 0.001
1 year max extension	2.6 (SD 3.6) 2.0 (IQR 5.0)	3.1 (SD 3.9) 3.0 (IQR 5.0)	4.1 (SD 4.4) 4.0 (IQR 6.0)	3.3 (SD 4.9) 2.5 (IQR 6.5)	0.03
2-year					
2 years OKS	43.1 (SD 6.9) 46.0 (IQR 5.0)	43.8 (SD 5.2) 46.0 (IQR 5.0)	43.0 (SD 5.6) 45.0 (IQR 6.0)	40.7 (SD 8.5) 44.0 (IQR 11.0)	0.003
2 years Tegner	3.1 (SD 1.3) 3.0 (IQR 2.0)	3.5 (SD 1.3) 3.0 (IQR 1.0)	3.1 (SD 1.1) 3.0 (IQR 1.0)	2.7 (SD 1.3) 3.0 (IQR 1.0)	< 0.001
2 years AKSS-O	92.8 (SD 11.9) 95.0 (IQR 6.5)	94.1 (SD 8.9) 97.0 (IQR 7.0)	93.8 (SD 8.1) 95.0 (IQR 6.0)	91.4 (SD 11.1) 95.0 (IQR 7.0)	0.048
2 years AKSS-F	87.6 (SD 14.7) 100.0 (IQR 20.0)	87.7 (SD 14.6) 90.0 (IQR 20.0)	84.8 (SD 15.9) 85.0 (IQR 30.0)	79.0 (SD 20.2) 80.0 (IQR 30.0)	< 0.001
2 years Charnley score	23.1% A 49.2% B 26.7% C	22.8% A 48.8% B 28.4% C	11.3% A 50.0% B 38.7% C	20.4% A 35.7% B 43.9% C	0.003
2 years max flexion	133.2 (SD 9.0) 134.0 (IQR 10.0)	129.7 (SD 8.6) 130.0 (IQR 11.0)	127.1 (SD 8.8) 128.0 (IQR 10.0)	120.0 (SD 12.2) 122.0 (IQR 14.0)	< 0.001
2 years max extension	2.0 (SD 3.7) 0.5 (IQR 3.0)	2.8 (SD 3.5) 2.0 (IQR 5.0)	3.5 (SD 3.9) 3.0 (IQR 5.0)	2.9 (SD 5.5) 1.0 (IQR 7.0)	0.08
5-year					
5 years OKS	43.1 (SD 6.6) 46.0 (IQR 6.0)	43.9 (SD 5.9) 46.0 (IQR 5.0)	41.0 (SD 7.8) 44.0 (IQR 9.0)	40.5 (SD 8.0) 44.0 (IQR 11.0)	< 0.001
5 years Tegner	3.1 (SD 1.4) 3.0 (IQR 2.0)	3.3 (SD 1.4) 3.0 (IQR 1.0)	2.8 (SD 1.3) 3.0 (IQR 1.0)	2.6 (SD 1.4) 3.0 (IQR 1.0)	< 0.001
5 years AKSS-O	93.6 (SD 8.3) 95.0 (IQR 7.0)	94.8 (SD 8.2) 98.0 (IQR 7.0)	91.2 (SD 11.8) 95.0 (IQR 7.5)	92.4 (SD 9.9) 95.0 (IQR 9.0)	0.01
5 years AKSS-F	84.3 (SD 17.0) 90.0 (IQR 20.0)	88.0 (SD 15.4) 100.0 (IQR 20.0)	80.1 (SD 16.3) 80.0 (IQR 30.0)	74.3 (SD 22.6) 80.0 (IQR 20.0)	< 0.001
5 years Charnley score	19.4% A 41.8% B 38.8% C	11.4% A 49.6% B 39.0% C	11.7% A 43.2% B 45.1% C	18.8% A 34.4% B 46.9% C	0.44
5 years max flexion	132.7 (SD 10.5) 134.5 (IQR 14.0)	129.4 (SD 8.1) 130.0 (IQR 10.0)	125.1 (SD 9.5) 125.0 (IQR 15.0)	121.8 (SD 10.5) 122.5 (IQR 13.0)	< 0.001
5 years max extension	2.5 (SD 4.2) 2.0 (IQR 5.0)	2.0 (SD 4.1) 1.0 (IQR 5.0)	2.2 (SD 3.8) 2.0 (IQR 5.0)	2.9 (SD 4.7) 0.0 (IQR 5.0)	0.56
10-year					
10 years OKS	42.0 (SD 5.6) 43.0 (IQR 11.0)	44.3 (SD 4.9) 46.0 (IQR 6.0)	40.1 (SD 9.7) 44.0 (IQR 9.0)	36.4 (SD 11.4) 38.0 (IQR 10.0)	0.04
10 years Tegner	2.5 (SD 1.1) 3.0 (IQR 1.0)	3.3 (SD 1.0) 3.0 (IQR 1.0)	2.3 (SD 1.3) 2.5 (IQR 2.0)	2.6 (SD 1.5) 3.0 (IQR 1.0)	0.08
10 years AKSS-O	90.5 (SD 12.1) 94.0 (IQR 10.0)	95.6 (SD 4.8) 96.5 (IQR 7.0)	87.5 (SD 17.5) 97.5 (IQR 29.0)	83.1 (SD 16.4) 93.0 (IQR 27.0)	0.09
10 years AKSS-F	80.9 (SD 16.0) 80.0 (IQR 30.0)	86.0 (SD 13.9) 90.0 (IQR 20.0)	67.9 (SD 11.8) 70.0 (IQR 12.5)	70 (SD 27.1) 75.0 (IQR 30.0)	0.007
10 years Charnley score	12.5% A 31.3% B 56.3% C	8.6% A 42.8% B 48.6% C	0.0% A 8.3% B 91.7% C	0.0% A 11.1% B 88.9% C	0.24
10 years max flexion	133.9 (SD 10.8) 133.0 (IQR 14.0)	128.5 (SD 9.7) 130.0 (IQR 17.0)	126.4 (SD 5.0) 125.0 (IQR 8.0)	125.6 (SD 9.1) 129.0 (IQR 10.0)	0.12
10 years max extension	2.9 (SD 4.9) 1.0 (IQR 7.0)	3.0 (SD 4.3) 3.0 (IQR 5.0)	2.6 (SD 3.1) 1.5 (IQR 5.0)	3.2 (SD 6.2) 5.0 (IQR 8.0)	0.99

PROMs were compared with the Kruskal–Wallis test, Charnley scores with the Chi-squared proportional test and flexion/extension with one-way ANOVA test

Comparing the postoperative OKS, Tegner, AKSS-O and between groups found that although there were some significant differences with the higher BMI groups having lower scores at all time points, these differences were generally small, except at 10 years. The AKSS-F was significantly lower in the higher BMI groups across all timepoints. However the highest BMI groups also had the lowest preoperative PROMs scores and on analysis of the differences in the score compared to preoperatively, high BMI groups actually had a greater gain in PROM score results when compared to the normal weight group (Table 4).

**Table 4:** Difference in mean preoperative and postoperative PROMs in different BMI groups

	BMI group			
	Normal	Overweight	Obese (class 1)	Obese (class 2)
1-year postoperatively				
OKS	16.1 (SD 8.5)	16.1 (SD 7.7)	17.0 (SD 9.4)	18.9 (SD 10.9)
Tegner	0.8 (SD 1.2)	0.7 (SD 1.3)	0.7 (SD 1.2)	0.7 (SD 1.2)
AKSS-O	26.8 (SD 16.8)	30.0 (SD 16.2)	28.4 (SD 18.5)	36.5 (SD 17.0)
AKSS-F	15.7 (SD 14.5)	14.2 (SD 16.2)	14.2 (SD 17.8)	12.9 (SD 18.6)
2-year postoperatively				
OKS	16.1 (SD 8.7)	17.0 (SD 7.4)	17.9 (SD 8.7)	20.3 (SD 10.5)
Tegner	0.7 (SD 1.1)	0.9 (SD 1.3)	0.8 (SD 1.2)	0.7 (SD 1.2)
AKSS-O	28.2 (SD 18.4)	30.8 (SD 15.5)	29.8 (SD 18.0)	36.3 (SD 17.6)
AKSS-F	14.2 (SD 15.3)	13.7 (SD 16.9)	13.2 (SD 18.2)	15.3 (SD 18.1)
5-year postoperatively				
OKS	16.7 (SD 9.1)	16.3 (SD 8.3)	16.1 (SD 9.8)	18.4 (SD 11.0)
Tegner	0.6 (SD 1.3)	0.7 (SD 1.4)	0.6 (SD 1.2)	0.8 (SD 1.6)
AKSS-O	31.5 (SD 16.9)	31.5 (SD 15.9)	31.9 (SD 20.0)	35.6 (SD 13.1)
AKSS-F	13.9 (SD 16.8)	12.9 (SD 18.2)	10.8 (SD 20.3)	8.9 (SD 19.0)

High mean flexion angles ranging between 119° and 134° were achieved in all BMI groups across all time points. The mean flexion angles decreased with increasing BMI with the flexion being about ten degrees less in the obese than normal weight (Table 3). The mean extension angles were between 2° and 4° at all time points for all BMI groups with no real differences between groups at all time points (Table 3).

There was a tendency for a greater proportion of patients with Charnley C scores in the higher BMI groups up to 5 years (Table 3). Subgroup analyses comparing the OKS of knees with Charnley scores of A and B compared to C in each BMI group are presented in Table 5. In all BMI groups at all time points the Charnley groups A and B scored higher than those of group C but these differences were minimal except in the obese groups where these were more marked.

**Table 5:** The OKS in different Charnley groups within each BMI group

BMI group	Charnley group	1 year	2 years	5 years
Normal	A and B	43.0 (SD 6.2) 45.0 (IQR 5.0)	44.0 (SD 6.1) 46.0 (IQR 3.0)	43.6 (SD 6.6) 46.5 (IQR 5.0)
	C	42.1 (SD 6.2) 45.0 (IQR 6.0)	41.1 (SD 8.1) 43.5 (IQR 9.5)	42.0 (SD 7.1) 45.0 (IQR 7.0)
Overweight	A and B	43.5 (SD 4.9) 45.0 (IQR 5.0)	44.5 (SD 4.7) 46.0 (IQR 5.0)	44.8 (SD 4.4) 46.5 (IQR 5.0)
	C	41.6 (SD 7.0) 44.0 (IQR 8.0)	42.7 (SD 5.6) 45.0 (IQR 7.0)	43.9 (SD 5.7) 46.0 (IQR 4.0)
Obese (class 1)	A and B	41.4 (SD 7.2) 44.0 (IQR 7.5)	43.6 (SD 5.8) 45.0 (IQR 4.0)	43.5 (SD 5.5) 46.0 (IQR 6.0)
	C	40.9 (SD 6.1) 43.5 (IQR 8.5)	41.9 (SD 5.5) 43.5 (IQR 6.0)	37.2 (SD 9.0) 40.5 (IQR 12.0)
Obese (class 2)	A and B	40.9 (SD 8.4) 44.0 (IQR 11.0)	43.1 (SD 7.1) 46.0 (IQR 6.0)	42.0 (SD 6.9) 46.0 (IQR 10.0)
	C	36.1 (SD 10.5) 39.0 (IQR 15.0)	37.6 (SD 9.5) 41.0 (IQR 15.0)	39.1 (SD 8.9) 42.0 (IQR 10.0)

## Discussion

This is the first study to investigate the effect of BMI on the outcomes of a cementless UKR. In all the BMI groups the 10-year survival for revision was between 96 and 98% and for reoperation was between 95 and 98%. This suggests that cementless UKR can be used in all BMI groups and that BMI should not be a contraindication.

When deciding whether to do a cementless UKR in an obese patient it is important to consider the main alternative which is a TKR. In our study cementless UKR revision rate did not increase with increasing BMI, whereas with TKR it does [1, 2, 17]. As a result in obesity the revision rate of cementless UKR may actually be less than TKR. For example in a meta-analysis of TKR studies of over 5 years the revision rate in obese patients was 5% whereas in our 10-year UKR study it was 3% [17]. In our study, the high levels of PROMs and range of movement in the obese groups, are better than those reported for TKR [25]. This is supported by evidence from many other sources that UKR provides better functional outcomes than TKR [40]. Analysis of large TKR data sets has found significantly higher rates of medical complication such as pulmonary embolism, deep vein thrombosis and infections in the obese compared to the non-obese [33, 38]. In contrast, an analysis of over 8,000 UKRs found no increased risk of any medical complications in obese patients for UKR surgery [36]. Additionally, the instrumentation for the Oxford knee works from the front and therefore is no more challenging in an obese patient, whereas a TKR is often technically more challenging in these patients. Taken together this not only supports the conclusion that obesity should not be a contraindication from cementless UKR but also suggests, if the indications are satisfied, that it is better to use a cementless UKR than a TKR for obese patients.

The finding that the revision rate of the cementless mobile-bearing UKR did not increase with obesity is somewhat counter-intuitive, as the increased loading would be expected to cause more damage to the implant, its fixation and the retained compartments. It might therefore be a type 2 error. This is however unlikely as in the two similar large studies of the cemented mobile-bearing UKR there was also no increase in revision rate in obesity [23, 24]. Furthermore in both the current study and the study of 2438 cemented mobile-bearing UKR [24] the group with the highest BMI had the lowest revision rate. One explanation is that obese patients have reduced levels of activity post-operatively, as demonstrated in this study by the lower Tegner and AKSS-F scores, and therefore subject their implants to fewer cycles [15]. However, this cannot be the complete explanation because the revision rate in TKR and some other designs of UKR increase in obesity [1, 2, 17, 42]. The reason is probably the design of the implant. The mobile bearing is fully congruent, minimising contact stresses and hence wear [35] and in this study, there were no problems due to wear. Additionally, as the bearing is mobile the loads at the bone-implant interfaces are predominantly compressive with minimal shear which reduces the risk of aseptic loosening [28] and in this study, there was only one case of aseptic loosening. Furthermore, the instrumentation is designed to accurately restore normal ligament function and tension, so restores normal knee kinematics and as a result the risk of disease progression laterally is very low. In this study, there were six cases of disease progression with more in the non-obese (*n* = 4) than in the obese (*n* = 2) groups. This is somewhat surprising as an elevated BMI is a potent risk factor for the development of knee osteoarthritis [3].

All BMI groups reported improvements in PROMs postoperatively at all timepoints compared to preoperatively. At the various time points higher BMI groups had slightly worse functional outcomes. However, the lowest preoperative scores were in the highest BMI groups. As a result the higher BMI groups generally had a greater improvement in scores compared to preoperatively. It is likely that in the obese patients, who tended to be young, the operations were not done until the symptoms were more severe due to concerns about the outcome following surgery.

The Charnley A and B knees had higher OKS than group C across all BMI groups. The differences were minimal in the normal and overweight groups but were more exaggerated in the obese groups. This may reflect how when more joints are disease affected this has a greater impact on functional outcomes in more obese individuals given these joints are burdened with increased loading.

This is the first study to investigate the effect of BMI on the mid to long outcomes of a cementless mobile-bearing unicompartmental knee replacement. Previous studies have investigated the effect of BMI on the outcomes of cemented UKRs and often report conflicting results. For mobile-bearing UKRs Malloy et al. [23], Murray et al. [24] and Pandit et al. [31] report that higher BMI did not affect survival rate although in some cases was associated with lower postoperative scores, although these groups tended to have the greatest increase in scores. However, both Nettour et al. [27] and Polat et al. [32] report higher revision rates in patients with raised BMIs. For fixed bearing UKRs Cavaignac et al. [7] and Woo et al. [41] did not find that BMI had any influence on implant survivorship. However Bonutti et al. [5] reported a 12% lower implant survival in BMIs > 35 kg/m^2^ compared to those < 35 kg/m^2^. More recently Xu et al. [42] report significantly worse 10-year implant survivals and functional scores for patients with BMI > 30.

The main strengths of this observational study are that it is a large prospective consecutive series of 1000 cementless Oxford UKRs using the recommended surgical indications with independent follow up. Additionally, several outcome measures were assessed pertaining to both implant survival and functional outcomes achieved. This information is not available in the joint registries. However the different BMI groups were not matched and therefore there were some differences in baseline characteristics of the different groups, such as the lower preoperative PROM scores and younger ages in the higher BMI groups. Additionally, this is a single centre study from the designer surgeons limiting its generalisability. However, if surgeons adhere to the recommended indications for the Oxford UKR their results are similar to those of the designer surgeons [13]. Therefore the study is generalisable provided the recommended indications are used. There were relatively small numbers of patients followed to 10 years, which is reflected by the wide confidence limits in revision and re-operation rates at this stage.

## Conclusions

This study found that the cementless mobile-bearing UKR had low reoperation and revision rates in all BMI groups and there were no significant differences between the groups. Although higher BMI groups had slightly worse functional outcomes, the improvement in function postoperatively tended to be better. The study, therefore, suggests that high BMI should not be considered to be a contraindication to the cementless mobile-bearing UKR.

## Abbreviations

AKSS-F: American Knee Society Functional Score
AKSS-O: American Knee Society Objective Score
AMOA: Anteromedial osteoarthritis
BMI: Body mass index
CI: Confidence intervals
IQR: Interquartile range
NJR: National Joint Registry of England, Wales, Northern Ireland and Isle of Man
OKS: Oxford Knee Score
PROM: Patient reported outcome measure
RCT: Randomised controlled trial
SD: Standard deviation
TKR: Total knee replacement
UK: United Kingdom
UKR: Unicompartmental knee replacement
